# A prediction nomogram for sodium-glucose co-transporter 2 inhibitors-induced diabetes ketoacidosis in non-surgical patients: a retrospective multicenter cohort study

**DOI:** 10.3389/fendo.2026.1915698

**Published:** 2026-09-09

**Authors:** Danxia Chen, Zongshuai Gao, Jian Wang, Yunxia Zhu, Jianwei Wan, Weijun Tang, Xiaojing Jiao, Yabin Ma, Feng Zhu, Xiucong Fan

**Affiliations:** 1 Department of Pharmacy, Shanghai East Hospital, Tongji University School of Medicine, Shanghai, China; 2 Department of Transfusion Medicine, Shanghai Sixth People’s Hospital Affiliated to Shanghai Jiao Tong University School of Medicine, Shanghai, China; 3 Department of Pharmacy, Shanghai Pudong New Area People’s Hospital, Shanghai, China; 4 Department of Laboratory Medicine, Shanghai East Hospital, Tongji University School of Medicine, Shanghai, China; 5 Department of Pharmacy, Shanghai University of Medicine and Health Science Affiliated Zhoupu Hospital, Shanghai, China; 6 Department of Pharmacy, Chinese Medicine Hospitals Changii Hui Autonomous Prefecture, Xinjiang, China; 7 Department of Critical Care Medicine, Shanghai East Hospital, Tongji University School of Medicine, Shanghai, China

**Keywords:** adverse drug reaction, in-hospital severity rates, nomogram, risk factors, sodium-glucose co-transporter 2 inhibitors-induced diabetes ketoacidosis

## Abstract

**Background:**

Sodium-glucose co-transporter 2 inhibitors (SGLT2i) are associated with a rare but serious risk of diabetic ketoacidosis (S-DKA). This study aimed to identify risk factors for S-DKA, develop a tool for its early detection, and assess the associated in-hospital severity.

**Methods:**

A retrospective, multicenter cohort study was conducted across four hospitals from September 2019 to June 2024. After screening 35,633 SGLT2i-prescribed inpatients, a 1:3 propensity score matching was applied. The study included 432 patients in the development cohort (2019-2023) and 128 in a validation cohort (2023-2024). Lasso regression and logistic regression were used to identify S-DKA risk factors and construct a nomogram. The primary endpoint for severity was the need for intensive care.

**Results:**

Six independent predictive factors for S-DKA were identified: female, indications with diabetes, high HbA1c levels, fasting, infection and low BMI. The prediction nomogram demonstrated excellent discrimination, with an area under the curve (AUC) of 0.920(95% CI: 0.888 -0.952) in the development cohort and 0.735 (95% CI: 0.644 -0.826) upon validation. The model was well-calibrated. Furthermore, patients with S-DKA required intensive care at a significantly higher rate compared to those without.

**Conclusion:**

S-DKA is associated with greater in-hospital severity, necessitating early identification. The developed nomogram provides a clinically valuable tool for predicting S-DKA risk, potentially aiding physicians in proactive patient management and improving outcomes.

## Introduction

Sodium-glucose co-transporter 2 inhibitors (SGLT2i) work by inhibiting glucose reabsorption in the renal proximal tubules, allowing glucose excretion in urine. Research has shown that SGLT2i can lower blood sugar, reduce blood pressure, promote weight loss, lower uric acid levels, and provide heart and kidney protective effects (1–4). Consequently, prescription rates have risen, bringing to light various adverse reactions, including reports of severe diabetes ketoacidosis (DKA) (5). From March 2013 to June 2014, the FDA received 20 FAERS reports related to SGLT2i-induced DKA(S-DKA), all requiring emergency hospital treatment (6). By May 2015, the reported cases had increased to 73, highlighting issues of delayed diagnosis due to insignificant blood sugar elevation (or euDKA) (7). Within a month, the FDA released two warnings and updated SGLT2i drug instructions to include information about S-DKA.

DKA is a rare and potentially life-threatening side effect characterized by metabolic acidosis and ketosis. Its causes in diabetes patients include hunger, low-calorie diet, fasting, alcoholism, pregnancy, chronic liver disease, insulin reduction, surgical stimulation, decreased liver and kidney function, etc (8–13). Some studies have explored SGLT2i-induced perioperative ketoacidosis in surgical patients (14, 15), attributing it to perioperative fasting and metabolic changes due to surgical stress (11, 16). However, so far, no research has validated the risk factors for non-surgical patient onset of S-DKA.

In order to recognize S-DKA better and earlier, physicians require an effective, simple, and intuitive tool. Nomograms, which are used to quantify risk factors, have been employed to predict the risk factors of various diseases, including predicting the occurrence and survival of gynecological tumors (17, 18), the incidence rate and prognosis of diseases related to newborns and obstetric surgery (19, 20), etc. In terms of predicting the risk of adverse drug reactions (ADRs), several studies have demonstrated the practicality of nomograms for immune checkpoint inhibitors (ICIs), tigecycline, and antiviral drugs (21–23).

In this study, we conducted a cohort study to analyze the clinical features and assess the risk factors for developing DKA in non-surgical patients using SGLT2i within internal medicine departments. The goal was to create a nomogram that predicts the risk of S-DKA. Furthermore, we examined the link between S-DKA and in-hospital severity rates among SGLT2i users. Our aim was to enable physicians to anticipate and mitigate these events by utilizing the nomogram for identifying S-DKA.

## Materials and methods

We adhered to the STROBE Statement for cohort studies in conducting this study.

No Artificial Intelligence Generated Content (AIGC) tools were used in any portion of the manuscript.

### Study design and setting

This study was designed as a retrospective, multicenter cohort study, which enrolled eligible patients at Shanghai East Hospital affiliated to Tongji University, Shanghai Sixth People’s Hospital, Shanghai Pudong New Area Zhoupu Hospital and Shanghai Pudong New Area People’s Hospital. The study incorporated 432 patients from the specified hospitals as the development group, with data collected between September 1, 2019, and August 31, 2023. Additionally, a validation group comprised 128 patients, and their data were collected from September 1, 2023, to June 30, 2024 in the four hospitals mentioned above. The corresponding inclusion and exclusion criteria of S-DKA and Non-S-DKA were also strictly followed in the validation group. The research design received approval from the Ethics Committees of Shanghai East Hospital affiliated to Tongji University (2024-183) with a waiver for informed consent. The registration number of this study in Chinese Clinical Trial Registry was MR-31-24-031290.

Eligible patients were those non-surgical ones who developed DKA after being treated with SGLT2i from September 1, 2019, to June 30, 2024. “Non-surgical” was defined as patients who did not undergo any surgical procedure, including emergency operations, daytime surgeries, or other invasive interventions prior to or during the index hospitalization. Exposure of SGLT2i was defined as both of pre-admission home medications and medications that were provided to patients during hospitalization. Exclusion criteria included: 1) patients without pre-medication lab results or medical history, 2) patients who received any surgical or invasive procedure prior to or during the current hospitalization and 3) patients with Type 1 Diabetes Mellitus. The time line was SGLT2i exposure (pre-admission and during hospitalization) → development of S-DKA → ICU admission (when clinically indicated). Pharmacists used the Naranjo Adverse Drug Reaction Probability Scale to evaluate the cause and effect of the adverse drug event (24). ADR scoring criteria: definite (≥9), probable (5 to 8), possible (1 to 4), and doubtful (≤0). Patients with scores ≥1 were classified as S-DKA, while Non-S-DKA (controls) included: 1) patients without DKA when treated with SGLT2i, and 2) patients who developed DKA when treated with SGLT2i but had a Naranjo Scale score of 0. All assessments were independently performed by two clinical pharmacists, with discrepancies resolved through discussion or third-party arbitration.

### Data collection

The collected data of enrolled patients were retrieved from both the Electronic Medical Record System and handwritten records. The data encompassed inpatient departments, age, gender, body mass index (BMI), smoking history, alcohol abuse, Charlson Comorbidity Index (CCI), indications for medication, fasting, insulin use, infection, glycosylated hemoglobin (HbA1C), liver and renal function, types of SGLT2i, medication combinations, and blood gas analysis indicators.

### Definition

DKA was defined as: 1) blood ketones≥3mmol/L or positive urine glucose with positive urine ketone body (more than ++); 2) arterial blood pH ≤ 7.3 and(or) bicarbonate <18.0 mmol/L (25); 3) hyperglycemia (blood glucose >13.9 mmol/L) or euglycemia (blood glucose ≤ 13.9 mmol/L). CCI (26) was a weighted index that takes into account the number and severity of comorbid diseases. It could be used to assess the severity of a patient’s comorbidities, with a point system consisting of 19 comorbidities. The CCI score was determined by adding up the weight of each comorbidity, which could be assigned as 1, 2, 3, or 6. For instance, a CCI score of 3 indicated three comorbidities each weighted as 1, or one comorbidity weighted as 1 and one as 2. Infection, liver, and renal function were defined based on diagnoses in medical records or receiving corresponding medical treatment during SGLT2i medication.

### Statistical analysis

R software (4.5.1) was utilized to perform a 1:3 propensity score matching (PSM) approach, which was as a tool to minimize confounding bias between S-DKA and non-S-DKA groups in both development cohort and validation cohort. The matched characteristics included age and CCI. In details, all eligible non-S-DKA patients identified from the screening cohort were included in the PSM candidate pool. Matching was then performed using a 1:3 nearest-neighbor algorithm without replacement, with a caliper width of 0.2. Group balance was assessed using standardized mean differences (SMD) for all PSM variables, with an SMD <0.10 indicating adequate balance. Missing data were minimal (<5% for all variables). Given the limited sample size (n=108), missing values were handled using multiple imputation by chained equations (MICE) with 5 imputations under the MAR assumption, including all predictors and the outcome in the imputation model. Continuous and categorical variables were imputed using predictive mean matching and logistic regression, respectively. Pooled estimates were derived using Rubin’s rules. Normal distribution was confirmed with the Kolmogorov–Smirnov test. Data following normal distribution were analyzed using t-tests and presented as means and standard deviations (SDs); non-normally distributed data were presented as medians with interquartile ranges (IQRs) and compared using Mann–Whitney U tests. Chi-square tests were used for frequency, rate, or constituent ratio. Binary logistic regression analysis was applied to screen independent predictors for S-DKA, after which variables with significant differences were further opted for Lasso regression model (p<0.05). To address potential multicollinearity among variables and further optimize the model, Lasso regression was employed for variable selection. Before performing Lasso regression, we assessed multicollinearity using variance inflation factors (VIF), and all VIF values were <5, indicating no substantial multicollinearity. Considering the limited number of positive events (108 cases) and to avoid model overfitting, the number of candidate predictors were restricted to enter Lasso regression based on the “one variable per 10-20 events” rule.

The penalty coefficient λ was determined by 10-fold cross-validation, selecting the largest λ within one standard error of the minimum cross-validated error (lambda.1se). All variables with non-zero coefficients at this λ value were retained for subsequent multivariable model development. Then multivariate logistic analysis was performed for variables above. Then predictors were used to create a nomogram using “rms package” in R software. The predictive accuracy of the model was evaluated using receiver operating characteristic (ROC) curve, area under the ROC curve (AUC), and calibration curve via pROC in R software. The prediction model was validated, and the 30-day in-hospital severity rate was compared using survival curves between the two groups.

## Results

### Baseline of the development and validation groups

**Figure 1:**
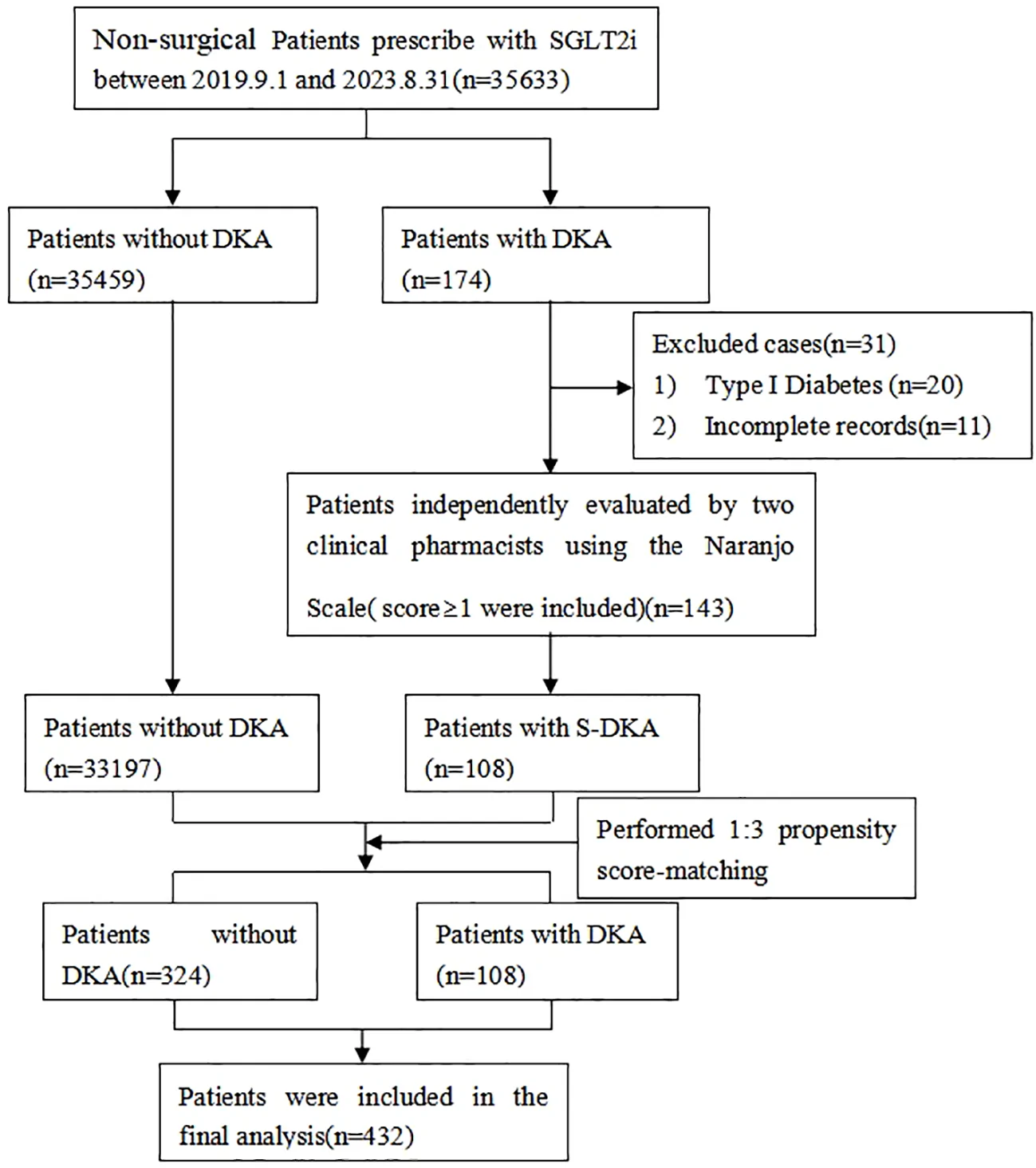
Flow chart of the study population enrollment. SGLT2i, Sodium-glucose co-transporter 2 inhibitors; DKA, severe diabetes ketoacidosis; S-DKA, SGLT2i-induced DKA.

From September 1, 2019, to August 31, 2023, a total of 35,633 hospitalized individuals who were administered SGLT2i were hospitalized in Shanghai East Hospital affiliated to Tongji University, Shanghai Sixth People’s Hospital, Shanghai Pudong New Area Zhoupu Hospital, and Shanghai Pudong New Area People’s Hospital. After accessing with Naranjo scale, 174 (0.49%) patients were found to have DKA, and 31 (17.82%) were excluded based on exclusion criteria. Two clinical pharmacists independently assessed the relationship between SGLT2i and DKA using the Naranjo Scale. The patients’ assessments were largely positive for the initial three items of the Naranjo scale, corresponding to a cumulative score of 3 to 4. (1. Were there previous conclusive reports on this reaction? Yes.+1/2. Did the adverse event appear after the suspected drug was administered? Yes.+2/3. Did the adverse reaction improve when the drug was discontinued or a specific antagonist was given? Yes.+1 or No. +0). After assessment, 35 (24.48%) patients were excluded due to the absence of a causal link with SGLT2i. Ultimately, 108 (62.07%) patients were confirmed to have been diagnosed with S-DKA. During the study, 33,197 SGLT2i users did not develop S-DKA. Following PSM, 108 cases and 324 controls were paired and included in the study as the development group. (**Figure 1**).

The baseline demographic and clinical characteristics before and after matching were detailed in **Table 1**. After PSM, the SMD for age and CCI were less than 0.10, indicating adequate balance between the S-DKA and non-S-DKA groups.

**Table 1:** Baseline characteristics of patients before and after propensity score matched analysis.

Variables		Before matched (n =33305)				After matched (n = 432)			
		S-DKA (n=108)	Non- S-DKA (n=33197)	p value	SMD	S-DKA (n=108)	Non- S-DKA (n=324)	p value	SMD
Median Age, years(IQR)		56(41-70)	66(60-74)	<0.001	-0.581	59(41-70)	59(41-70)	0.967	-0.004
CCI, n(%)	0	0	1195(3.6)	<0.001	-0.432	0	1(0.3)	0.531	0.085
	1	8(7.3)	7038(21.2)			8(7.3)	22(6.8)		
	2	51(46.9)	6075(18.3)			51(46.9)	151(46.6)		
	3	22(20.8)	8565(25.9)			22(20.8)	68(21.0)		
	≥4	27(25.0)	10324(30.9)			27(25.0)	82(25.3)		

DKA, severe diabetes ketoacidosis; S-DKA, SGLT2i-induced DKA; CCI, Charlson Comorbidity Index.

In this study, the data of 432 patients were used to create a nomogram. The validation group comprised 128 inpatients (32 cases and 96 controls) receiving SGLT2i medication between September 1, 2023, and June 30, 2024 from the four hospitals. Baseline characteristics of the development and validation groups were presented in **Table 2**.

**Table 2:** Baseline characteristics of the development and validation groups.

Variables		Development group			Validation group		
		S-DKA (n=108)	Non-S-DKA (n=324)	p value	S-DKA (n=32)	Non-S-DKA (n=96)	p value
Demographic data							
Males, n (%)		48 (44.4)	201 (62.0)	0.041	13 (40.6)	69 (71.9)	0.001
BMI, kg/m^2^ (IQR)		23.5 (20.5-25.8)	25.5 (22.3-27.8)	0.783	24.26 (22.79-25.76)	25.0 (22.3-27.5)	0.018
CCI, n (%)	0	0	1 (0.3)	0.531	0	16 (16.7)	<.001
	1	8 (7.3)	22 (6.8)		0	9 (9.4)	
	2	51 (46.9)	151 (46.6)		19 (59.4)	9 (9.4)	
	3	22 (20.8)	68 (21.0)		9 (28.1)	10 (10.4)	
	≥4	27 (25.0)	82 (25.3)		4 (12.5)	52 (54.2)	
Smoking history, n (%)		20 (18.5)	80 (24.7)	0.128	6 (18.8)	24 (25.0)	0.13
Drinking history, n (%)		16 (14.8)	45 (13.9)	0.617	2 (6.3)	23 (24.0)	0.23
Comorbidity							
Fasting, n (%)		17 (15.7)	15 (4.6)	<.001	1 (3.1)	2 (2.1)	0.106
Infection, n (%)		46 (42.6)	38 (11.7)	<.001	10 (31.2)	12 (12.5)	<.001
Liver dysfunction, n (%)		13 (12.0)	45 (13.9)	0.432	1 (3.1)	5 (5.2)	0.332
Kidney dysfunction, n (%)		22 (20.4)	86 (26.5)	0.264	4 (12.5)	26 (27.1)	0.035
Physical examination							
HbA1C,% (IQR)		10.52 (8.93-12.0)	7.53 (6.12-8.75)	0.038	10.34 (8.95-11.88)	8.37 (6.43-9.48)	0.078
Blood sugar (real time),mmol/L (IQR)		17.65 (10.82-22.37)	8.09 (5.83-9.87)	<.001	17.39 (11.60-18.41)	8.73 (5.83-9.33)	0.001
SBP, mmHg (IQR)		140 (130-148)	136 (127-152)	0.243	140 (128-145)	143 (130-149)	0.094
DBP, mmHg (IQR)		93 (82-101)	90 (80-102)	0.342	92 (79-99)	89 (78-103)	0.086
SGLT2i							
Dapagliflozin, n (%)		87 (80.6)	270 (83.3)	0.075	25 (78.1)	71 (74.0)	0.742
Engagliflozin, n (%)		18 (16.7)	31 (9.6)		5 (15.6)	17 (17.7)	
Kagliflozin, n (%)		2 (1.9)	21 (6.5)		0	8 (8.3)	
Etogliflozin, n (%)		1 (0.9)	1 (0.3)		2 (6.3)	0	
Indications for SGLT2i							
Diabetes, n (%)		108 (100.0)	203 (62.7)	<.001	30 (93.8)	77 (80.2)	0.023
Other Diseases, n (%)		0 (0.0)	121 (37.3)	2 (6.3)	19 (19.8)		
Concomitant use of other diabetes medications							
Biguanides, n (%)		37 (34.3)	38 (11.7)	0.038	8 (25.0)	9 (9.4)	0.789
Simultaneous use of one diabetes drug, except biguanides, n (%)		17 (15.7)	37 (11.4)	2 (6.2)	14 (14.6)		
Simultaneous use of two diabetes drugs, except biguanides, n (%)		26 (24.1)	85 (26.2)	12 (37.5)	23 (24.0)		
Simultaneous use of three diabetes drugs, except biguanides, n (%)		11 (10.2)	25 (7.7)	3 (9.4)	12 (12.5)		
Insulin, n (%)		43 (39.8)	123 (38.0)	0.726	9 (28.1)	36 (37.5)	0.031

DKA, severe diabetes ketoacidosis; S-DKA, SGLT2i-induced DKA; BMI, Body Mass Index; CCI, Charlson Comorbidity Index; SBP, Systolic Blood Pressure; DBP, Diastolic Blood Pressure;.

Concomitant use of other diabetes medications refers to pre-admission home medications that patients were taking prior to hospitalization.

### Predictive factors of S-DKA

**Figure 2:**
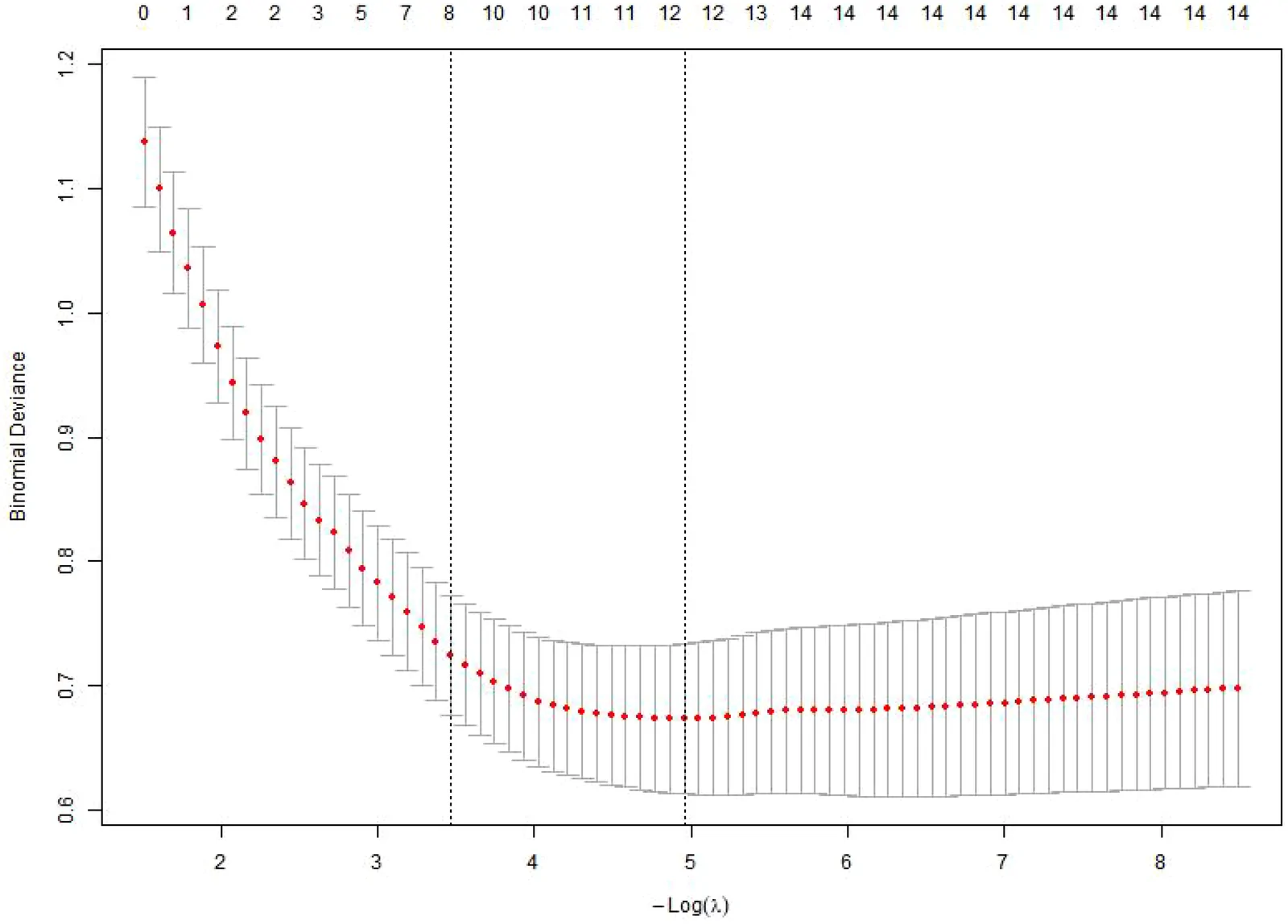
LASSO cross validation diagram.

**Figure 3:**
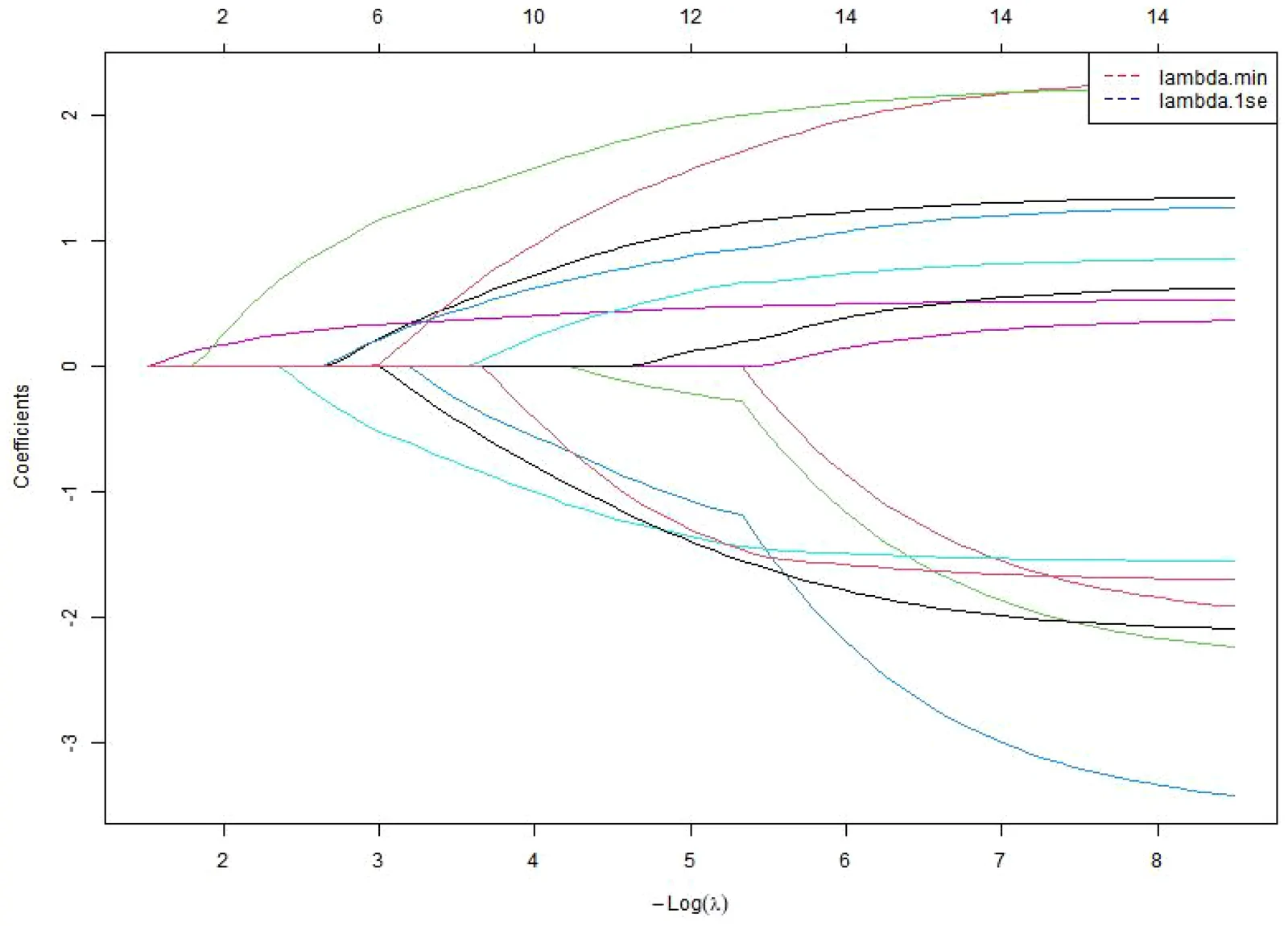
LASSO regression path diagram.

The clinical features of the development group were analyzed using univariate, lasso and multivariate logistic regression to predict S-DKA. **Table 3** showed the univariate analysis, variables associated with S-DKA included female, indications with diabetes, high HbA1c levels, fasting, infection, low BMI and concomitant use of diabetes drugs. LASSO cross-validation plots (**Figure 2**) and regression path diagrams were generated (**Figure 3**). Lasso analysis in **Table 4** illustrated that at the optimal penalty coefficient λ of 0.031, seven variables with non-zero coefficients were identified, including the 7 factors contribute significantly to model the construction. In the multivariate analysis, independent predictors of S-DKA were identified as female (OR 3.490, 95%CI 1.676 ~ 7.270, p <0.001), indications with diabetes (OR 6.228, 95%CI 1.204 ~ 32.215, p=0.029), HbA1C (OR 1.723, 95%CI 1.443 ~ 2.057, p <0.001), fasting (OR 11.672, 95%CI 2.446 ~ 55.706, p=0.002), infection (OR 7.936, 95%CI 3.594 ~ 17.521, p<0.001), BMI(18.5-23.9, OR 0.105, 95%CI 0.013 ~ 0.828, p<0.032; 24.0-27.9, OR 0.083, 95%CI 0.010 ~ 0.665, p<0.019; ≥28.0, OR 0.023, 95%CI 0.002 ~ 0.221, p=0.001). The results of multivariate logistic regression analysis were shown in **Table 5**.

**Table 3:** Univariate regression analysis.

Variables	β	S.E	Z	*P*	OR (95%CI)
Sex, n (%)					
Male					1.000 (Reference)
Female	0.808	0.249	3.245	**0.001**	2.244 (1.377 ~ 3.655)
BMI					
<18.5					1.000 (Reference)
18.5-23.9	-1.884	0.715	-2.636	**0.008**	0.152 (0.037 ~ 0.617)
24.0-27.9	-1.967	0.719	-2.733	**0.006**	0.140 (0.034 ~ 0.573)
≥28.0	-2.420	0.765	-3.161	**0.002**	0.089 (0.020 ~ 0.399)
Indications for SGLT2i					
Other Disease					1.000 (Reference)
Diabetes	3.213	0.727	4.423	**<0.001**	24.857 (5.985 ~ 103.241)
Fasting					
No					1.000 (Reference)
Yes	1.360	0.401	3.390	**<0.001**	3.898 (1.775 ~ 8.561)
Infection					
No					1.000 (Reference)
Yes	1.925	0.281	6.862	**<0.001**	6.858 (3.957 ~ 11.887)
HbA1C (%)	0.576	0.073	7.925	**<0.001**	1.779 (1.542 ~ 2.051)
Concomitant use of other diabetes medications					
None					1.000 (Reference)
Biguanides	1.920	0.369	5.200	**<0.001**	6.824 (3.308 ~ 14.073)
Simultaneous use of one diabetes drug, except biguanides	1.084	0.422	2.572	**0.010**	2.957 (1.294 ~ 6.755)
Simultaneous use of two diabetes drugs, except biguanides	0.745	0.360	2.069	**0.039**	2.107 (1.040 ~ 4.269)
Simultaneous use of three diabetes drugs, except biguanides	1.167	0.501	2.327	**0.020**	3.211 (1.202 ~ 8.577)

SGLT2i, Sodium-glucose co-transporter 2 inhibitors; BMI, Body Mass Index; HbA1C, Glycated Hemoglobin A1c. Bold values are variables with significant differences (p<0.05).

**Table 4:** Lasso regression coefficient.

Variable	Coefficient	Optimal λ
(Intercept)	-4.9113795	0.03123981
Infection	1.3699357	
Indications	-0.75132	
Fasting	0.5131328	
Sex	0.481016	
Concomitant use of other diabetes medications	0.429101	
HbA1C	0.3671295	
BMI	-0.2309536	

Categorical and ordinal variables in the Lasso regression were coded as follows: Sex (Male = 0, Female = 1); Indications (Diabetes = 0, Other Diseases = 1); Fasting (No = 0, Yes = 1); Infection (No = 0, Yes = 1).

**Table 5:** Multivariate logistic regression analysis.

Variables	β	S.E	Z	*P*	OR (95%CI)
Intercept	-6.461	1.502	-4.300	**<0.001**	0.002 (0.000 ~ 0.030)
Sex					
Male					1.000 (Reference)
Female	1.250	0.374	3.339	**<0.001**	3.490 (1.676 ~ 7.270)
BMI					
<18.5					1.000 (Reference)
18.5-23.9	-2.250	1.052	-2.139	**0.032**	0.105 (0.013 ~ 0.828)
24.0-27.9	-2.492	1.064	-2.343	**0.019**	0.083 (0.010 ~ 0.665)
≥28.0	-3.761	1.148	-3.276	**0.001**	0.023 (0.002 ~ 0.221)
Indications for SGLT2i					
Other Disease					1.000 (Reference)
Diabetes	1.829	0.838	2.181	**0.029**	6.228 (1.204 ~ 32.215)
Fasting					
No					1.000 (Reference)
Yes	2.457	0.797	3.081	**0.002**	11.672 (2.446 ~ 55.706)
Infection					
No					1.000 (Reference)
Yes	2.071	0.404	5.126	**<0.001**	7.936 (3.594 ~ 17.521)
HbA1C	0.544	0.090	6.023	**<0.001**	1.723 (1.443 ~ 2.057)

SGLT2i, Sodium-glucose co-transporter 2 inhibitors; BMI, Body Mass Index; HbA1C, Glycated Hemoglobin A1c. Variables with significant differences(p<0.05)

### Development and validation of nomogram

**Figure 4:**
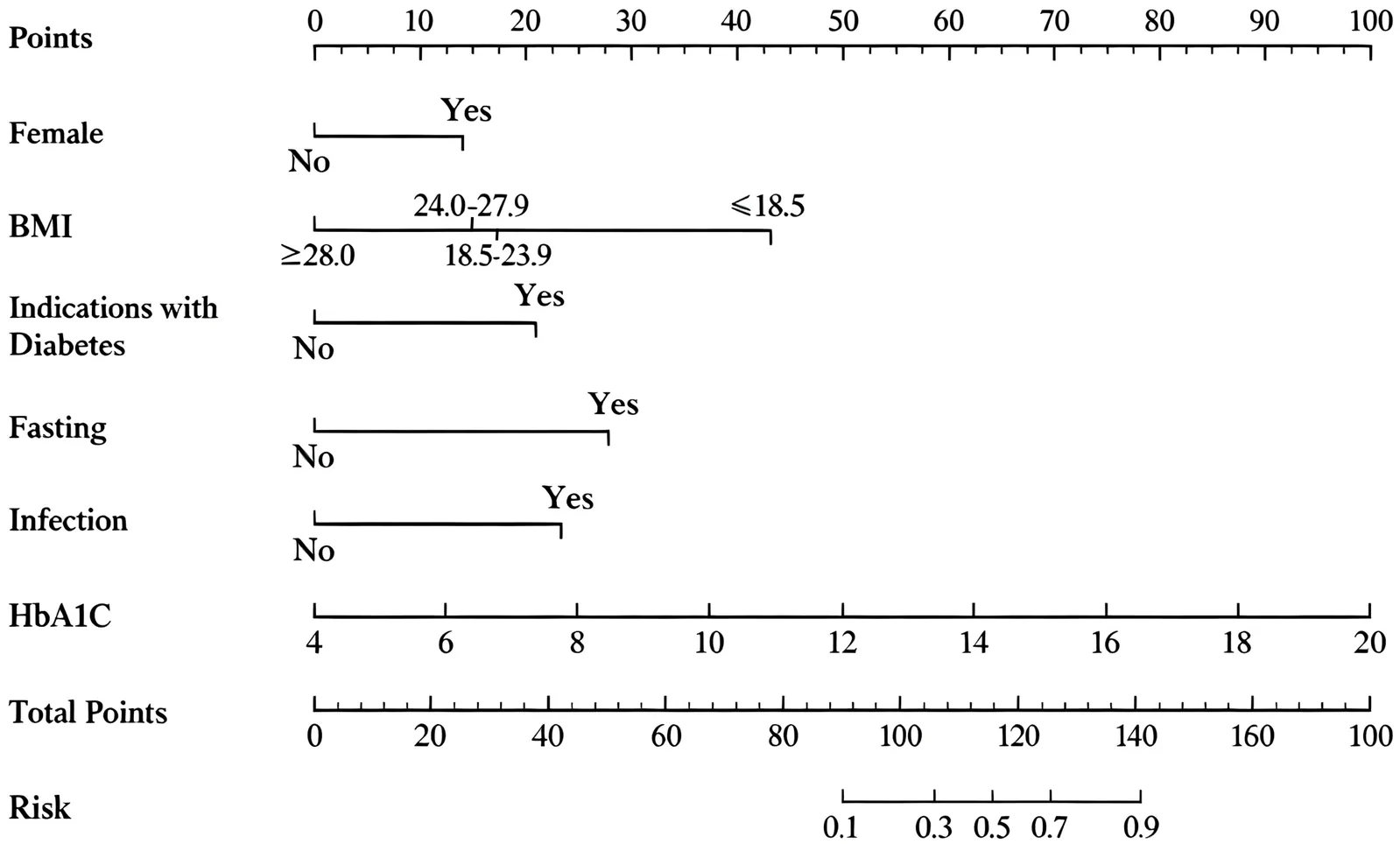
Nomogram model for predicting the occurrence of S-DKA. BMI, Body Mass Index; HbA1C, Glycated Hemoglobin A1c.

The model presented a nomogram containing 6 predictors from multivariate logistic regression analysis (**Figure 4**). This nomogram used independent predicting variables alongside weighted scores. HbA1C was a continuous variable whereas female, indications with diabetes, fasting, infection and BMI are categorical. Total points were calculated as the sum of scores in the nineth row. Predicted risk value was obtained by matching total scores, which predicted the probability of S-DKA occurrence. The S-DKA probability ranged from 0.1 to 0.9. A higher total score denoted a higher incidence rate of S-DKA.

*Logit(P) = −6.4607 + 1.2500×I(sex=female) −2.2504×I(BMI = 18.5-23.9) −2.4922 ×I(BMI = 24.0-27.9) −3.7607×I(BMI≥28.0)+1.8291×I(indications without diabetes)+2.4572×I(fasting=yes)+2.0714×I(infection=yes)+0.5441×HbA1C*.Where I(*) denoted the indicator function, which took a value of 1 if the condition was met and 0 otherwise. The predicted probability of S-DKA occurrence was subsequently derived using the standard logistic transformation:

For example, a female patient with diabetes, BMI 24.5 kg/m², on SGLT2i, non-fasting, with infection, and HbA1c 10.0% would have a Logit(P) of −0.1905 and a predicted S-DKA probability of 45.2%.

**Figure 5:**
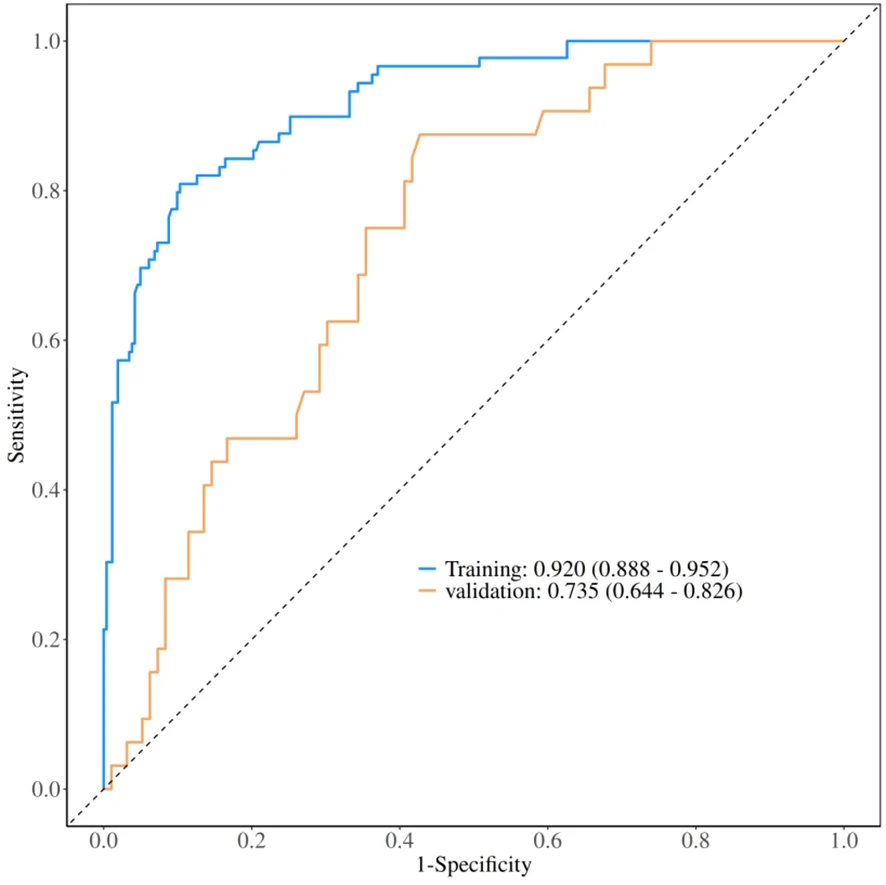
ROC curves in the development and validation groups.

**Figure 6:**
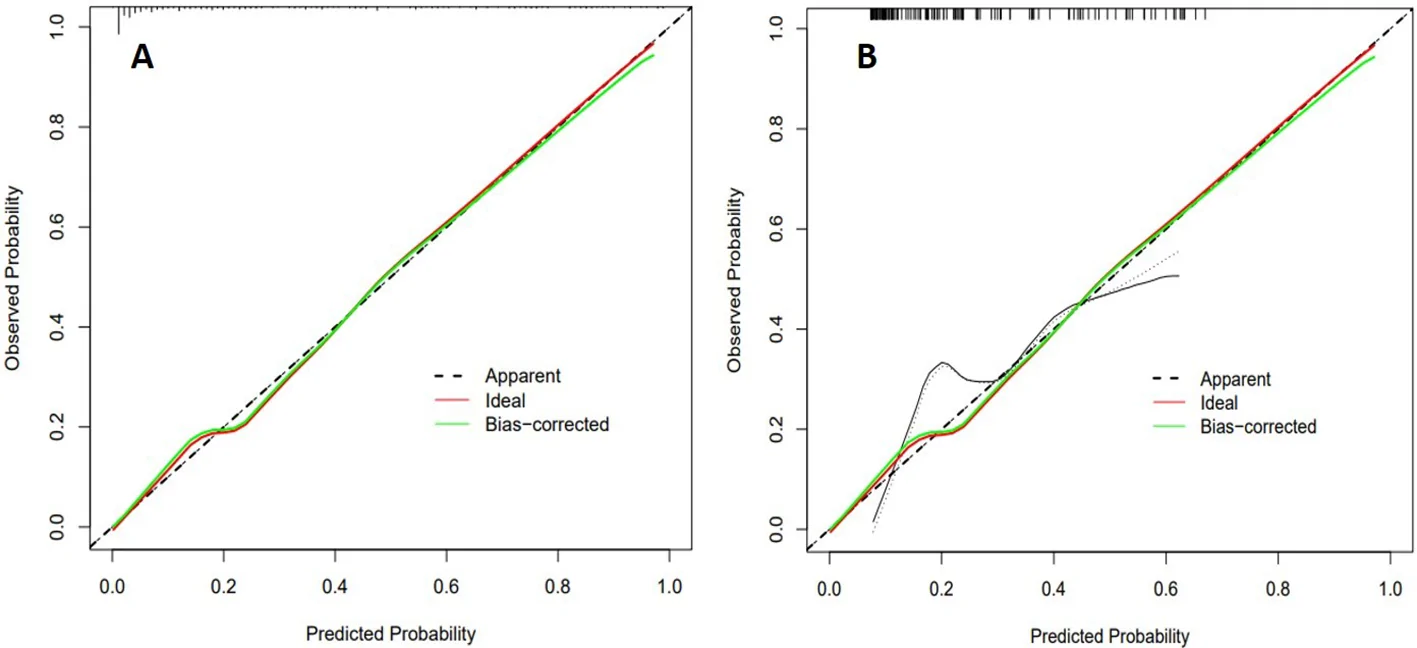
Calibration curves for predicting the probability of S-DKA. **(A)** Development group **(B)** Validation group.

**Figure 7:**
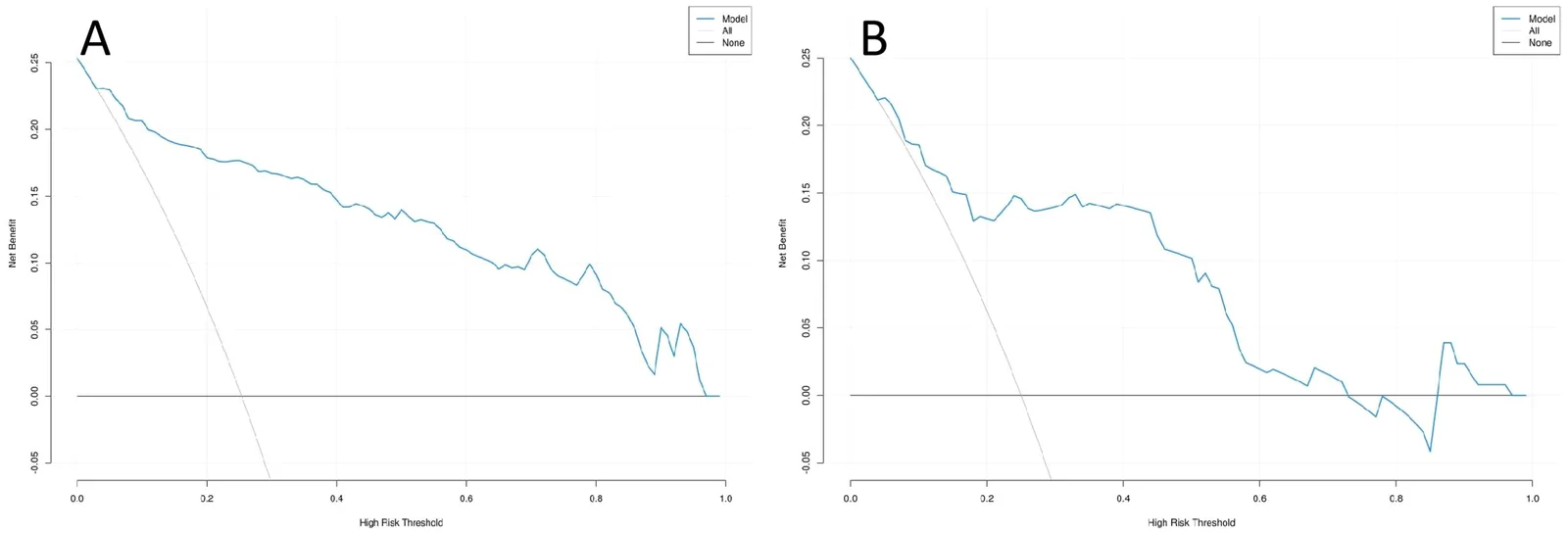
Decision curve analysis for predicting the probability of S-DKA. **(A)** Development group **(B)** Validation group.

AUC of the nomogram was 0.920 (95%CI 0.888-0.952) in the development group and 0.735 (95%CI 0.644-0.826) in the validation group (**Figure 5**). Furthermore, the calibration curve of the nomogram for predicting the risk of S-DKA was close to the ideal diagonal line, which demonstrated good agreement in this study (**Figure 6**).The application of DCA to assess the clinical utility of the nomograms presented promising results in both development group and validation group (**Figure 7**).

### The association between S-DKA and severity rate

**Figure 8:**
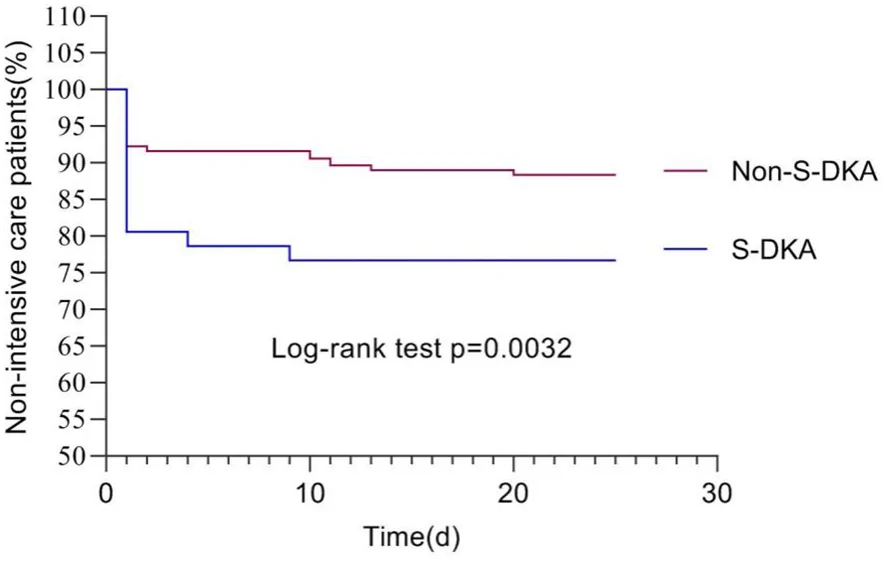
Severity rate (probability of non-intensive care) for the S-DKA and non-S-DKA group. S-DKA, SGLT2i-induced severe diabetes ketoacidosis.

A survival curve was used with the log-rank test to analyze the connection between S-DKA and severity rate. The curve indicated that patients with S-DKA have a higher likelihood of intensive care (p < 0.05) (**Figure 8**), showing an increased severity of the disease.

## Discussion

The mechanism behind S-DKA was certain. It involved an increase in the production of free fatty acids, which were then converted into ketone bodies, as well as the reabsorption of ketone bodies by renal tubules (27, 28). Currently, there hasn’t been a systematic study on the occurrence of S-DKA. Liu J et al. (29) indicated in a review that SGLT2i were linked to a 2.13-fold higher risk of DKA in T2DM patients compared to placebo or other non-insulin hypoglycemic drugs, with an absolute occurrence rate of 3/1000 per patient year. Colacci M et al. (30) demonstrated in a meta-analysis that the absolute rate of DKA among patients given an SGLT2i varied from 0.6 to 2.2 events per 1,000 patient years. The incidence rate in this study was around 3/1000 per patient year, falling within the range of the two aforementioned studies. Since S-euDKA patients often exhibit atypical clinical symptoms with normal or slightly elevated blood sugar levels (31), which almost entirely manifest as a distinct ADR of SGLT2i, the actual occurrence is often underestimated. As a result, the construction of a predictive model is urgently necessary.

In this study, we examined 14 predictive variables and ultimately determined 6 predictive factors. Advanced age was not among the 14 factors because this factor has been confirmed to elevate the incidence of DKA in previous researches and incorporated in predictive risk models (32, 33). CCI represents patients’ overall illness severities. To reduce demographic bias, we included age and CCI in PSM. In this study, lower BMI was significantly associated with a higher susceptibility to DKA. This result aligns with the Asia-Pacific expert consensus, which identifies lower BMI as a susceptibility factor for euDKA (34). Furthermore, low BMI may serve as a surrogate marker for insulin deficiency or poor nutritional status, thereby indirectly increasing the risk of DKA (35).

Fasting has been recognized as a DKA risk factor, including euDKA, in various studies (36, 37). The pharmacological mechanism of SGLT2i might explain this phenomenon by directing energy from carbohydrates to adipose tissue, which leads to increased ketone production. This is especially evident during fasting or when following a low-carb/ketogenic diet, where insufficient carbohydrate intake further encourages SGLT2i to drive metabolism towards lipids, potentially causing DKA. Gender is commonly factored into disease risk models, and in this study, females were more likely to experience S-DKA than males. While no study has indicated differences in DKA incidence rates between genders, we hypothesize that, due to the fasting and diet-related mechanism of DKA, females may be more inclined to adopt dietary lifestyles, indirectly contributing to this phenomenon.

Studies have revealed that infection is the primary cause of DKA, accounting for up to 30% (38, 39). The percentage of DKA cases due to infection in the UK is 45% (40), whereas in India, this rate is as high as 73.3% (41). Infections from any part of the body can lead to DKA, with urinary tract infections and pneumonia being the most commonly reported causes (42). In line with the aforementioned data, cases in the development cohort show that 42.6% (46/108) of S-DKA cases were associated with infection, with pneumonia and urinary tract infections being the most prevalent (**Supplementary Figure 1**). These results were easy to understand, exposed to the environments of high blood sugar and disrupted nutrient metabolism, immune function is prone to decline, making body susceptible to pathogen invasion (43). While the presence of infection can worsen patient conditions and extend hospitalization periods, it also increases the risk of systemic inflammatory reactions, thereby raising the mortality rate associated with DKA. The predictive nomogram provides physicians with a tool for early DKA detection, ensuring that infections are scarcely overlooked.

For the factor of indications with diabetes, no patients without diabetes were found to have S-DKA (**Table 2**; **Supplementary Figure 2**). This aligns with several trials for heart failure treatment using SGLT2i, where no instances of DKA occurred in non-diabetic patients (44–47). However, a recent study of Mahesh MU et al. (48) revealed two non-diabetic patients with DKA after SGLT2i treatment for heart failure. Although S-DKA in non-diabetic patients is rare, given the extensive use among heart failure and renal patients, such instances may persist (49). The findings above suggest indications with diabetes may be a possible high-risk signal. However, due to the limited number of cases, its predictive value requires cautious interpretation, and it should not be considered a core strong predictor in the model. Thus, a precise risk model utilizing six factors with specific weights meets the need for early identification of high-risk groups, as opposed to relying solely on medication indications for predicting S-DKA probability.

HbA1C plays a crucial role in screening for diabetes, evaluating blood sugar control, assessing effectiveness, and monitoring progress in follow-up (50). It serves as the standard for evaluating long-term blood glucose control. Research demonstrates that the HbA1C levels of nearly all DKA patients have significantly increased. Effective medical treatment can reduce the occurrence of DKA (51). A study by OE C et al. (52) suggests that both T1DM and T2DM patients show significantly elevated HbA1C levels in cases of ketoacidosis. Previous studies have shown that as HbA1C levels rise, blood HBA levels also increase, and elevated HBA levels are a key diagnostic factor for DKA, which may explain the connection between HbA1C and S-DKA (53).

While the validation cohort was derived from the same four centers but from a different time period, which serves as a strengthened internal validation, notable baseline differences were observed between the development and validation cohorts. For instance, the distribution of CCI scores differed significantly between the two cohorts (p < 0.001), which may have contributed to the decline in AUC from 0.920 in the development cohort to 0.735 in the validation cohort. An AUC of 0.735 remains within the acceptable range (generally considered > 0.70) for clinical prediction models, despite the suggestion of potential overfitting in the development model. Importantly, the calibration curve in the validation cohort remained close to the ideal diagonal line, indicating good model calibration. Besides, we have noted that 2024 ADA/International Consensus (54, 55) adopted a glucose threshold of <200 mg/dL (11.1 mmol/L) for euDKA, which was different from the definition criteria of this study. However, our retrospective study commenced in January 2024, and the diagnostic criteria we adopted were based on the Chinese clinical practice standards in effect at the time of study inception (the 2021 CDS Guideline). Therefore, subsequent studies will adopt the updated 2024 consensus criteria, more rigorous threshold of ADR and aim to include more centers with a prospective setting, in order to conduct external validation and incorporate more potentially predictive variables.

To date, this is the first cohort study in a real-world multicenter setting to create a nomogram for predicting the occurrence of DKA and assessing the likelihood of intensive care in patients treated with SGLT2i among the non-surgical population. Several outpatient-based prediction models have attempted to quantify S-DKA risk. Fralick et al. (56, 57)developed a risk score prediction model in a population-based cohort, identifying prior DKA, insulin use, and HbA1c >9% as key predictors, with an overall AUC of approximately 0.63 in derivation and 0.66 in external validation. Notably, their model did not include acute precipitants such as fasting or infection, and the authors explicitly acknowledged that time-varying variables were not captured, potentially leading to risk underestimation in acute settings. This study addresses this gap with a nomogram that integrates both acute phase predictors (fasting, infection) and baseline factors (female sex, diabetic indication, HbA1c, low BMI), reflecting real-world inpatient practice. Our model shows excellent discrimination (AUC 0.920 in development, 0.735 in temporal validation), substantially outperforming prior outpatient tools. The developed nomogram demonstrated good predictive performance and included a suitable number of predictive indicators, each of which is easily obtained in clinical practice. Regarding the severity of S-DKA, previous studies have indicated that these patients require a longer time to recover compared to those with DKA from other causes (58). The survival curve in this study showed a higher risk of intensive care probability in comparison to patients with non-S-DKA, highlighting the importance of early identification of high-risk populations for S-DKA. Collectively, our nomogram offers a pragmatic, bedside-applicable tool tailored for the inpatient non-surgical setting, which complements existing outpatient risk awareness and filling an unmet clinical need.

However, there are a few limitations that need to be addressed. Firstly, cases of S-euDKA may be underestimated due to insufficient recognition of symptoms and the relatively low severity of the patients’ illness. Therefore, given the relatively low incidence rate of S-DKA resulting in few positive cases, we are unable to analyze additional variables including significant test indicators such as random blood glucose and stress events, etc. Besides, as a retrospective study, several potential confounders were not collected in this study, including patients’ dietary patterns (e.g., low-carbohydrate or ketogenic diets), duration of SGLT2i use prior to admission, and time of drug discontinuation. These factors have been implicated in S-DKA risk and should be incorporated in future prospective studies. Secondly, we acknowledge that a higher threshold (e.g., “probable” ADR, score ≥5) would be more stringent. However, for patient safety, rechallenging or increasing the dose to confirm diagnosis is unethical when an adverse reaction is suspected. We deliberately chose a score of 1-4 (“possible”) to maximize sensitivity in capturing potential S-DKA cases, as used in previous studies (59, 60). We note that this may overestimate causality, and future studies should consider sensitivity analyses with higher Naranjo thresholds. Lastly, in this study, ICU admission was used as the primary indicator of S-DKA severity. While we attempted to control for potential confounding by matching on age and CCI, this measure may still be influenced by patients’ overall disease burden and institutional admission practices, which could introduce residual bias. Moreover, since all S-DKA events occurred exclusively in patients with diabetic indications, with no events in the non-diabetic group, the limited number of events precluded robust multivariable analysis to assess whether the baseline indication independently predicts in-hospital severity (e.g., ICU admission). Therefore, while we consider ICU admission a statistically meaningful and clinically relevant endpoint for comparing overall acuity between groups, the relationship between SGLT2i indication and severity outcomes beyond S-DKA risk remains an open question requiring further prospective investigation. Future studies should also incorporate more objective criteria to refine severity assessment.

In conclusion, this study focused on the occurrence of S-DKA in patients receiving non-surgical treatment and identified six predictors of S-DKA. As a result, a nomogram was developed to forecast the likelihood of S-DKA. Finally, individuals with S-DKA were found to have a higher chance of requiring intensive care compared to those without S-DKA, highlighting the significance of early identification using this risk model.

## Data Availability

The original contributions presented in the study are included in the article/**Supplementary Material**. Further inquiries can be directed to the corresponding authors.

## Supplementary material

Supplementary Figure 1 Distribution of infections in patients with S-DKA (n=108). S-DKA, SGLT2i-induced severe diabetes ketoacidosis.

Supplementary Figure 2 Patients using SGLT2i for different indications(n=108), CKD: Chronic Kidney Dysfunction.
