# Supplementary material for: A prediction nomogram for sodium-glucose co-transporter 2 inhibitors-induced diabetes ketoacidosis in non-surgical patients: a retrospective multicenter cohort study
Source: Front Endocrinol (Lausanne). 2026 Sep 9;17:1915698. doi: 10.3389/fendo.2026.1915698 (PMC13597255; doi:10.3389/fendo.2026.1915698)
Supplement: Supplementary file 1 [file SupplementaryFile1.pdf]

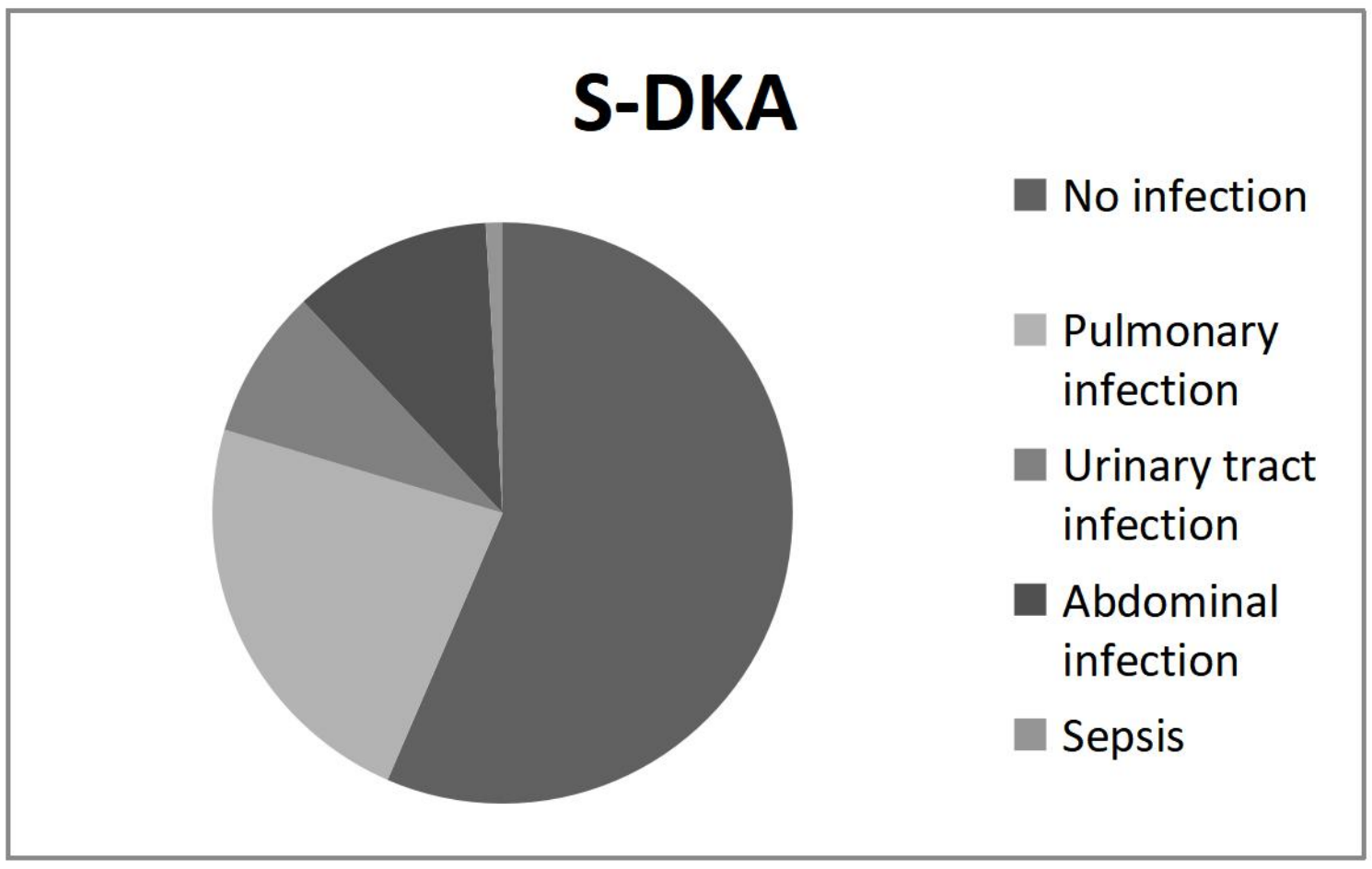

Figure S1. Distribution of infections in patients with S-DKA (n=108)

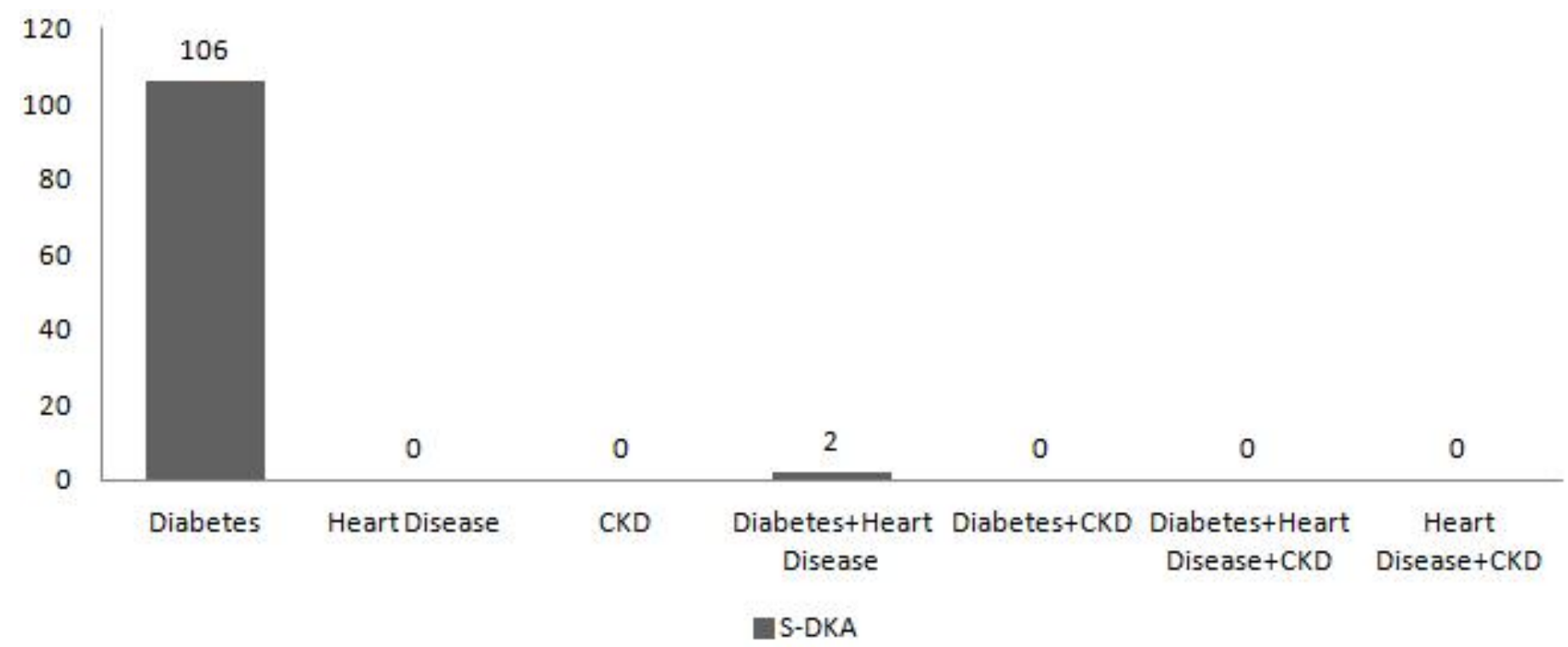

Figure S2. Patients using SGLT2i for different indications(n=108)，CKD：Chronic Kidney Dysfunction
